# An Algebraic Derivation of Chao's Estimator of the Number of Species in a Community Highlights the Condition Allowing Chao to Deliver Centered Estimates

**DOI:** 10.1155/2014/847328

**Published:** 2014-11-11

**Authors:** Jean Béguinot

**Affiliations:** ^1^Société d'Histoire Naturelle du Creusot, 12 rue des Pyrénées, 71200 Le Creusot, France; ^2^Biogéosciences, Université de Bourgogne, 21000 Dijon, France

## Abstract

Anne Chao proposed a very popular, nonparametric estimator of the species richness of a community, on the basis of a limited size sampling of this community. This expression was originally derived on a statistical basis as a lower-bound estimate of the number of missing species in the sample and provides accordingly a minimal threshold for the estimation of the total species richness of the community. Hereafter, we propose an alternative, algebraic derivation of Chao's estimator, demonstrating thereby that Chao's formulation may also provide *centered* estimates (and not only a lower bound threshold), provided that the sampled communities satisfy a specific type of SAD (species abundance distribution). This particular SAD corresponds to the case when the number of unrecorded species in the sample tends to decrease exponentially with increasing sampling size. It turns out that the shape of this “ideal” SAD often conforms approximately to the usually recorded types in nature, such as “log-normal” or “broken-stick.”. Accordingly, this may explain why Chao's formulation is generally recognized as a particularly satisfying nonparametric estimator.

## 1. Introduction

Estimating the total species richness within large communities of species, using only samplings limited in sizes, is a common, long standing challenge which has elicited numerous procedures of estimations.

For a few decades, a series of so-called nonparametricestimators provide elegant and convenient solutions to this question. These new estimators are ordinarily simple format formulations which, moreover, require no specific assumption regarding the statistical distribution of the abundances of species (which, thus, makes these formulations “nonparametric”).

Within this category of formulations, Anne Chao proposed a very popular nonparametric expression, which actually stands among the most commonly used estimators of the total species richness of a sampled community.

Let Δ be the number of species that are missed by (i.e., unrecorded within) the limited sampling of a large community of species. Then, according to Chao's formulation [1],

(1) $$\begin{array}{c} \Delta =\frac{f_{1}^{2}}{2f_{2}} \end{array}$$

with *f*_1_ and *f*_2_ as the numbers of species encountered only* once* and* twice*, respectively, in the sample.

This expression was later generalised [2] as

(2) $$\begin{array}{c} \Delta ={\left[\frac{f_{1}^{x}}{\left(x!f_{x}\right)}\right]}^{\left(1/(x-1)\right)} \end{array}$$

with *f*_*x*_ standing for the number of species recorded *x* times in the sample.

These expressions, derived on a statistical basis, provide a lower-bound estimate of the number Δ of missing species in a sampled community [1, 2]. That is, Chao's formulation is expected to provide only a minimal threshold (in the statistical sense) for the estimated species richness. Yet, several decades of practice (especially in the field of ecological purposes and biodiversity surveys) call for placing Chao's expression among the most valuable and reliable estimators [3–7] since, in many occasions, this expression may well appear to provide approximatelycentered rather than only lower-bound estimates. In short, although designed conceptually as a lower-bound evaluation, Chao's formulation may, nonetheless, fairly often provide rather centered estimates in the common practice.

Hereafter, we address this apparent paradox and propose new insights and argumentations, issued from an alternative, algebraic derivation of the originally statistically derived formulation by Chao.

## 2. The Specific Condition Allowing an Alternative, Algebraic Derivation of the “Chao” Formulation

We demonstrate (see Appendix A for mathematical details) that the general expression of Chao's estimator, established originally on a statistical basis, may also admit, alternatively, an algebraic derivation, leading to exactly the same expression as the statistically derived formulation Δ = [*f*_1_^*x*^/(*x*!*f*_*x*_)]^(1/(*x*−1))^.

Yet, while the statistical derivation of Chao's formulation requires no particular restriction (“nonparametric”), the algebraic derivation, implies a particular shape for the expected decrease of the proportion Δ/*S* of missing (i.e., unrecorded) species when the sample size *N* increases. In fact, as demonstrated in Appendix A, this asymptotic decrease should conform to a negative exponential:

(3) $$\begin{array}{c} \frac{\Delta }{S}=\exp\left(-kN\right) \end{array}$$

with

(i) *“S*” as the species richness, that is, the total (unknown) number of species of the community,
(ii) “*k*” as a constant,
(iii) “*N*” as the sample size, that is, the number of individuals recorded in the sample.

In turn, this particular form of the decrease of the number of missing species with enlarging sample sizes constrains the shape of the species abundance distribution (the “SAD,” that is, the distribution of species abundances when species are conventionally ranked by decreasing order of abundance).

According to (3), the number “*r*” of recorded species in the sample is

(4) $$\begin{array}{c} r=S-\Delta =S\left(1-\exp\left(-kN\right)\right). \end{array}$$

Let *k*′ be the number of individuals belonging to the less abundant species among the “*r*” species recorded in the sample (i.e., the species of rank “*r*” when species are ranked by decreasing order of abundance). Then, the relative abundance *a*_*r*_ of the species of rank *r* is expected to be inversely proportional to *N*, as

(5) $$\begin{array}{c} a_{r}=\frac{k^{\prime }}{N}. \end{array}$$

According to (4) and (5),

(6) $$\begin{array}{c} r=S\left[1-\exp\left(-\frac{kk^{\prime }}{a_{r}}\right)\right]. \end{array}$$

According to the sample size *N*, every species may be called to play the role of the less abundant species within the sample (since, by continuously decreasing the size *N* of the sample, each species of the community [including, at last, the most common] would successively play the role of the least abundant species in the sample).

Therefore, (6) stands the same for any species of any rank “*i*” in the SAD:

(7) $$\begin{array}{c} i=S\left[1-\exp\left(-\frac{kk^{\prime }}{a_{i}}\right)\right]. \end{array}$$

**Figure 1:**
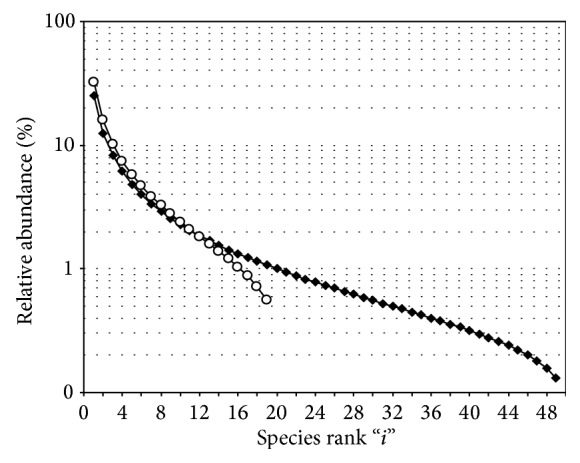
Typical shape of the “SAD” when the number Δ of unrecorded species is decreasing as a negative exponential of the sample size. The sum of abundances is normalised at 100% (*open circles*: total species richness = 20;* black diamonds*: total species richness = 50).

This equation thus describes the shape of the species abundance distribution (*a*_*i*_ = *f*(*i*)) when the proportion of unrecorded species in a sample is exponentially decreasing with the sample size, that is, the shape of the species abundance distribution which conditions the validity of the algebraic derivation of Chao estimator.  Figure 1 provides examples of the corresponding shapes of the SAD.

## 3. The Resulting Restrictive Condition Which Allows the “Chao” Formulation to Become a Centered Estimator of Species Richness

As the algebraic derivation is deterministic by essence, it therefore provides a centered estimate of Δ and of the resulting total species richness *S*, instead of being only a lower-bound estimate, as is the case in the nonparametric context. As mentioned above, the algebraic derivation of Chao's formulation requires that the sampled community satisfies, at least approximately, the particular type of SAD defined by (7) and illustrated at Figure 1. This restrictive condition is the “price” to be paid for the more accurate estimate, namely, the loss of the strict “nonparametric” character of the statistically based conception.

Yet, this condition assigned to the shape of the distribution of species abundances might not be so restrictive in practice, at least as a first approximation. Reasons for this may be as follows:

(a) an asymptotic decrease to zero of Δ with *N* (equation (3)) seems logical and intuitive, because aiming to estimate the total number of species in a community implicitly requires that this number does exist and might be actually reached progressively with sampling size *N* increasing continuously;
(b) among the different types of accumulation curves with such an asymptotic evolution, the negative exponential answer of Δ to increasing sampling size is, admittedly, one among the most simple, robust, and seemingly common [8, 9];
(c) the sigmoidal shape of the prescribed SAD (equation (7) and Figure 1) is not so far from the most classically referred empirical types, broken-stick and log-normal distributions [10]. Yet, a strict conformity is not expected a priori with any empirical models. For example, the equation *i* = *f*(*a*_*i*_) for the SAD corresponding to a broken-stick distribution is *i* ≈ *S* · exp⁡(−*S* · *a*_*i*_) [10], which is formally different from (7).

Accordingly, it is no real surprise that Chao's formula often approaches a strictly centred estimate, in spite of being only a lower-bound estimate in all generality.

This would explain why, in ecological practice in particular, Chao's formulation is yet considered one of the more accurate and reliable estimators of the total species richness within partially sampled communities.

As mentioned in particular by Gotelli (personal communication), a trend would remain for Chao's estimates to increase somehow when a series of sampling of growing sizes are extracted from the same community instead of remaining ideally stable on average. This, however, is not necessarily contradictory to preceding arguments but should certainly result from residual discrepancy between the real SAD and the ideal model described by (7) and exemplified at Figure 1.

## Appendix

### A. An Alternative, Algebraic Derivation of the Chao 1 Estimator

Consider a community of species, actually containing an unknown total number “*S*” of species.

Let *p*_*i*_ be the probability of occurrence (assimilated to the relative abundance) of species “*i*” within this community and let *N* be the sample size, that is, the number of individuals recorded in the sample.

The estimated number of species that escape recording during sampling of the community is Δ_(*N*)_:

(A.1) $$\begin{array}{c} \Delta_{\left(N\right)}=\Sigma_{i}{\left(1-p_{i}\right)}^{N} \end{array}$$

with Σ_*i*_ as the operation summation extended to the totality of the “*S*” species “*i*” (either* recorded *or* not*).

The number *f*_*x*_ of species recorded *x times* in the sample is then, according to the binomial distribution:

(A.2) fx=N!/X!N−x!Σi1−piN−xpix=CN,xΣi1−piN−xpix.

#### A.1. Deriving *f*_*x*_ as a Function of Δ_(*N*)_

The number *f*_*x*_ of species recorded *x times* in the sample may be derived as a function of Δ_(*N*)_.

(i) Consider

(A.3) f1=NΣi1−piN−1pi=NΣi1−piN−11−1−pi according  to  A.2=NΣi1−piN−1 −NΣi1−piN−11−pi=NΣi1−piN−1−NΣi1−piN.

According to (A.1), it becomes as follows: *f*_1_ = *N*(Δ_(*N* − 1)_ − Δ_(*N*)_) = −*N*(Δ_(*N*)_ − Δ_(*N*−1)_).

As appropriate sampling requires a size *N* ≫ 1, the difference (Δ_(*N*)_ − Δ_(*N*−1)_) can be likened to the corresponding derivative (∂Δ_(*N*)_/∂*N*). Then,

(A.4) $$\begin{array}{c} f_{1}=-N\left(\frac{\partial \Delta_{\left(N\right)}}{\partial N}\right)=-N\Delta_{\left(N\right)}^{\prime }, \end{array}$$

where Δ_(*N*)_′ is the first derivative of Δ_(*N*)_ with respect to *N*. Thus,

(A.5) $$\begin{array}{c} f_{1}=-N\Delta_{\left(N\right)}^{\prime }\left(=-C_{N,1}\Delta_{\left(N\right)}^{\prime }\right). \end{array}$$

Similarly,

(ii) consider

(A.6) f2=CN,2Σi[1−piN−2pi2] according  to  (A.2)  =CN,2Σi1−piN−21−1−pi2  =CN,2Σi1−piN−2−Σi1−piN−21−pi2  =CN,2Σi1−piN−2−Σi1−piN−21−pi1+pi  hhhhhh−Σi1−piN−21−pi1+pi  =CN,2Σi1−piN−2−Σi1−piN−11+pi  =CN,2ΔN−2−ΔN−1−f1N         according  to  (A.1)  and  (A.2)  =CN,2−ΔN−1′−f1N=CN,2−ΔN−1′ + ΔN′since  f1=−NΔ(N)′ (cf.  equation  (A.5)).  =CN,2∂ΔN′∂N  =NN−12∂2ΔN∂N2=NN−12ΔN′′,

where Δ_(*N*)_′′ is the second derivative of Δ_(*N*)_ with respect to *N*. Thus,

(A.7) $$\begin{array}{c} f_{2}=\left[\frac{N\left(N-1\right)}{2}\right]\Delta_{\left(N\right)}^{\prime \prime }=C_{N,2}\Delta_{\left(N\right)}^{\prime \prime }. \end{array}$$

(iii) Consider

(A.8) f3=CN,3Σi1−piN−3pi3       which,by  the  same  process,yields:=CN,3Σi1−piN−3−Σi1−piN−2−Σi[1−piN−2pi2]    −Σi1−piN−2pi      −Σi[1−piN−2pi2]=CN,3ΔN−3−ΔN−2−f1∗N−1−2f2NN−1         according  to  (A.1)  and  (A.2),

where *f*_1_^*^ is the number of singletons that would be recorded in a sample of size (*N* − 1) instead of *N*.

According to (A.5) and (A.7),

(A.9) $$\begin{array}{c} f_{1}^{*}=-\left(N-1\right)\Delta_{\left(N-1\right)}^{\prime }=-C_{N-1,1}\Delta_{\left(N-1\right)}^{\prime },\\ f_{2}=\left[\frac{N\left(N-1\right)}{2}\right]\Delta_{\left(N\right)}^{\prime \prime }=C_{N-1,2}\Delta_{\left(N\right)}^{\prime \prime }, \end{array}$$

where Δ_(*N* − 1)_′ is the *k*th derivate of Δ_(*N*)_ with respect to *N*, at point (*N* − 1). Then,

(A.10) f3=CN,3[(ΔN−3−ΔN−2)+ΔN−1′−ΔN′′]=CN,3[−ΔN−2′+ΔN−1′−ΔN′′]=CN,3[ΔN−1′′−ΔN′′]=CN,3−∂ΔN′′∂N=CN,3−∂3ΔN∂N3=CN,3ΔN′′′,

where Δ_(*N*)_′′′ is the third derivative of Δ_(*N*)_ with respect to *N*. Thus,

(A.11) $$\begin{array}{c} f_{3}=-C_{N,3}\Delta_{\left(N\right)}^{\prime \prime \prime }. \end{array}$$

(iv) Generalising this approach to calculate the number *f*_*x*_ of species recorded *x times* in the sample:

(A.12) fx=CN,xΣi[1−piN−xpix] (according  to  (A.2))=CN,xΣi1−piN−x1−1−pix=CN,xΣi1−piN−x−Σi1−piN−x1−pix=CN,xΣi1−piN−x−Σi1−piN−x1−piΣjpij   −Σi1−piN−x1−piΣjpij

with Σ_*j*_ as the summation from *j* = 0 to *j* = *x* − 1. It becomes as follows:

(A.13) fx=CN,xΣi1−piN−x−Σi1−piN−x+1Σjpij=CN,xΣi1−piN−x−Σk(Σi1−piN−x+1pik)    −Σi1−piN−x+1    −Σk(Σi1−piN−x+1pik)

with Σ_*k*_ as the summation from *k* = 1 to *k* = *x* − 1; that is,

(A.14) fx=CN,xΔN−x−ΔN−x+1−Σkfk∗CN−x+1+k,kaccording  to  A.1  and  A.2,

where *C*_(*N*−*x*+1+*k*),*k*_ = (*N* − *x* + 1 + *k*)!/*k*!/(*N* − *x* + 1)! and *f*_*k*_^*^ is the number of species recorded *k* times during a sampling of size (*N* − *x* + 1 + *k*) (instead of size *N*).

The same demonstration, which yields previously the expression of *f*_1_^*^ above (see (A.9)), applies for the *f*_*k*_^*^ (with *k* up to *x* − 1) and gives

(A.15) $$\begin{array}{c} f_{k}^{*}={\left(-1\right)}^{k}\left(C_{\left(N-x+1+k\right),k}\right)\Delta_{\left(N-x+1+k\right)}^{\left(k\right)}, \end{array}$$

where Δ_(*N*−*x*+1+*k*)_^(*k*)^ is the *k*th derivate of Δ_(*N*)_ with respect to *N*, at point (*N* − *x* + 1 + *k*). Then,

(A.16) fx=N!/x!N−x!×ΔN−x−ΔN−x+1−Σk−1kΔN−x+1+kk,

which finally yields

(A.17) fx=N!/x!N−x!−1x∂ΔNx−1∂N=N!/x!N−x!−1x∂xΔN∂Nx.

That is,

(A.18) $$\begin{array}{c} f_{x}={\left(-1\right)}^{x}C_{N,x}\Delta_{\left(N\right)}^{\left(x\right)}, \end{array}$$

where Δ_(*N*)_^(*x*)^ is the *x*th derivative of Δ_(*N*)_ with respect to *N*, at point *N*.

A relationship is thus derived between the series of the numbers *f*_*x*_ of species recorded *x* times and the series of derivatives Δ_(*N*)_^(*x*)^ at order *x* for Δ_(*N*)_:

(A.19) $$\begin{array}{c} f_{0}=\Delta_{\left(N\right)}=C_{N,0}\Delta_{\left(N\right)},\\ f_{1}=-C_{N,1}\Delta_{\left(N\right)}^{\prime },\\ f_{2}=C_{N,2}\Delta_{\left(N\right)}^{\prime \prime },\\ f_{3}=-C_{N,3}\Delta_{\left(N\right)}^{\prime \prime \prime },\\ f_{x}={\left(-1\right)}^{x}C_{N,x}\Delta_{\left(N\right)}^{\left(x\right)}. \end{array}$$

These relations will serve as the basis for deriving the formulation of Δ as a function of the *f*_*x*_.

#### A.2. Deriving a Generalised Expression for the “Chao” Formulation

Let us consider now the case when the number of missing species Δ_(*N*)_ conforms (or is close) to a negative exponential with respect to the sampling size *N*. Accordingly, Δ_(*N*)_ would thus verify the following series of differential equations:

(A.20) $$\begin{array}{c} \frac{\Delta_{\left(N\right)}}{\Delta_{\left(N\right)}^{\prime }}=\frac{\Delta_{\left(N\right)}^{\prime }}{\Delta_{\left(N\right)}^{\prime \prime }}=\frac{\Delta_{\left(N\right)}^{\prime \prime }}{\Delta_{\left(N\right)}^{\prime \prime \prime }}=\cdots =\frac{\Delta_{\left(N\right)}^{\left(x-1\right)}}{\Delta_{\left(N\right)}^{\left(x\right)}} \end{array}$$

that yields Δ_(*N*)_/Δ_(*N*)_^(*x*)^ = (Δ_(*N*)_/Δ_(*N*)_′)^*x*^ and then Δ_(*N*)_ = [(Δ_(*N*)_′)^*x*^/Δ_(*N*)_^(*x*)^]^1/(*x*−1)^, where Δ_(*N*)_′, Δ_(*N*)_′′, Δ_(*N*)_′′′, and Δ_(*N*)_^(*x*)^ are the first, second, third, and *x*° derivatives of Δ_(*N*)_ with respect to *N*.

As Δ_(*N*)_′ = −(*f*_1_/*N*) and Δ_(*N*)_^(*x*)^ = (−1)^*x*^ *f*_*x*_/*C*_*N*,*x*_ (see (A.5) and (A.18)), it follows for Δ_(*N*)_ that

(A.21) $$\begin{array}{c} \Delta_{\left(N\right)}={\left(C_{N,x}\left[\frac{f_{1}^{x}}{N^{x}f_{x}}\right]\right)}^{\left(1/(x-1)\right)}. \end{array}$$

Note that *C*_*N*,*x*_/*N* ^*x*^ is ~1/*x*! since, in practice, *x* remains, by far, quite smaller than *N*. Accordingly,

(A.22) $$\begin{array}{c} \Delta_{\left(N\right)}={\left[\frac{f_{1}^{x}}{\left(x!f_{x}\right)}\right]}^{\left(1/(x-1)\right)}. \end{array}$$

In particular,

(A.23) ΔN=f0=f122f2 [the  genuine  Chao's  formulation]=f136f30.5=f1424f40.33⋯.
